# Characteristics and predictors of severe outcomes of COVID‐19 cases presenting to the emergency department of a major Australian referral hospital: A record linkage study

**DOI:** 10.1111/1742-6723.70040

**Published:** 2025-04-10

**Authors:** Katrina Boldt, Nectarios Rose, Sandra Ware, Michael M Dinh, Kishor Kumar Paul, Yvonne Ho, David J Muscatello

**Affiliations:** ^1^ School of Population Health University of New South Wales Sydney New South Wales Australia; ^2^ Greater Sydney Helicopter Emergency Medical Service NSW Ambulance Sydney New South Wales Australia; ^3^ Emergency Department, RPA Green Light Institute Royal Prince Alfred Hospital Sydney New South Wales Australia; ^4^ NSW Biostatistics Training Program NSW Ministry of Health Sydney New South Wales Australia

**Keywords:** COVID‐19, emergency department, healthcare data, hospitalisation, linked data

## Abstract

**Objective:**

To describe the characteristics, outcomes and predictors of a severe outcome of patients presenting with a SARS‐CoV‐2 infection to the ED of a major urban referral hospital in New South Wales, Australia, from January 2020 through February 2022.

**Methods:**

Linked healthcare and death registration records were used and included any person assigned a diagnosis potentially related to an acute respiratory infection in the ED and that had a linked positive COVID‐19 detection. Logistic regression was used to determine predictors of a severe outcome (ICU admission or death) within 28 days.

**Results:**

Of 2081 included COVID‐19 patients, 238 (11.4%) had a severe outcome within 28 days of arrival at the ED. Among adults, the odds of a severe outcome increased with age, although the rate of increase in odds within age groups was statistically significant only in 30–64‐year‐olds (4% per year of age; confidence interval [CI] 2–6). Ambulance arrival (odds ratio [OR] 2.85; CI 1.76–4.78), higher triage urgency (category 1 or 2 compared with 4 or 5: OR 8.63; CI 4.41–18.12), and presentation during the pre‐Delta (OR 6.18; CI 3.59–10.66) and Delta (OR 3.64; 95% CI 2.49–5.35) variant periods (compared with Omicron) were independently associated with increased risk of a severe outcome.

**Conclusion:**

Age, ambulance arrival, higher triaged urgency, and presentation earlier in the pandemic were predictors of a severe COVID‐19 outcome. Aged care measures and prioritising vaccination of older persons and aged care workers may have reduced severe outcomes in the elderly.

## Graphical abstract

Using linked healthcare, SARS‐CoV‐2 notification and death registration records for patients with COVID‐19 presenting to a major Australian hospital, we found that arrival by ambulance, higher triaged urgency and presenting earlier in the pandemic were independent predictors of intensive care admission or death at 28 days. Although the risk of a severe outcome increased with age, it did not increase further after age 65 years.

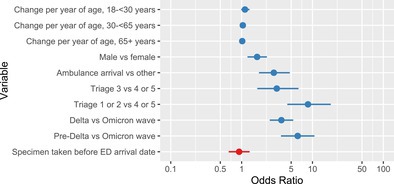

**Key findings**

- Older age, higher urgency at triage, arrival by ambulance and presenting earlier in the pandemic were associated with more severe outcomes.
- A vaccination strategy prioritising vulnerable populations and associated workers, along with improved treatment and changing variant severity, may have contributed to a reduced frequency over time of severe outcomes following ED presentation in Australia.
- Non‐pharmaceutical control measures in aged care facilities and, later, vaccination may have attenuated the increasing risk of a severe outcome with increasing age in the oldest population.

## Introduction

In December 2019, multiple pneumonia cases of unknown cause occurred in Wuhan, China.^1^ The causative virus was eventually isolated as SARS‐CoV‐2 and common symptoms of initial cases included fever, cough, myalgia or fatigue,^1^ and complications such as acute respiratory distress syndrome.^1^, ^2^ The disease was soon named Coronavirus Disease 2019 (COVID‐19).^3^

Australia recorded its first case on 25 January 2020. As cases increased, public health measures were introduced, and emergency management plans were enacted.^4^ Community transmission was largely controlled to mid‐2021 through domestic and international border closures, hotel quarantine for international and interstate arrivals, movement restrictions, and intensive test‐trace‐isolate‐quarantine strategies, and public health and social measures such as hand washing and mask wearing.^5^

Prior to the introduction of COVID‐19 vaccination in February 2021, almost all of the population of the state of New South Wales (NSW, population > 8 million) remained susceptible to infection.^6^ In June 2021, an outbreak of the Delta variant began,^7^ which was more transmissible than earlier variants,^8^ and which led to the re‐introduction of state‐level control measures. In December 2021, when >90% vaccine coverage was reached in NSW, control measures were relaxed.^9^ However, that coincided with the arrival of the Omicron variant, which was able to evade existing immunity^10^ and community transmission increased.^11^

There is a dearth of information on the experience of Australian EDs in relation to COVID‐19, and documenting the experience may assist preparedness for future pandemics. Using linked healthcare and administrative records, which provide information on both pre‐ and post‐ED care and outcomes, the present study aimed to describe the characteristics and outcomes of COVID‐19 patients presenting to a major tertiary referral hospital in New South Wales until early 2022 and to determine predictors of a severe outcome.

## Methods

### Study design and setting

The study was a retrospective descriptive and analytical study of patients presenting to the ED of Royal Prince Alfred (RPA) Hospital, a major urban, public, tertiary referral hospital in New South Wales (NSW), Australia. The ED serves adults and paediatric patients and received 81 774 presentations in the 2018/19 financial year.^12^

### Data sources

The study data were drawn from the Pandemic and Epidemic Assessment of Risk using Linked data (PEARL) resource, a research repository of de‐identified ED presentation records from the NSW ED Data Collection (EDDC) database for patients with an ED diagnosis potentially related to an acute respiratory infection. For each ED patient, the database also includes probabilistically linked hospital inpatient admission records from the NSW Admitted Patient Data Collection (APDC), COVID‐19 infections from the Notifiable Conditions Information Management System (NCIMS), and deaths from the state death registry.^13^

The inclusion criteria for the PEARL database is any ED presentation in the EDDC allocated a primary ED diagnosis of an acute respiratory infection, fever, cough, a non‐specific infection, breathing problems (non‐asthma) or sepsis. The ED diagnosis classification used at the selected hospital was SNOMED Clinical Terminology (CT) and included SNOMED CT IDs are available in Appendix S1.

### Study inclusion criteria

Inclusion for the present study required ED presentation to the study hospital from 1 January 2020 through 28 February 2022, an ED diagnosis included in the PEARL database, and a confirmed or probable SARS‐CoV‐2 infection. Infections were obtained from the linked NCIMS database which included detections by reverse transcription polymerase chain reaction (RT‐PCR) assay (confirmed) or, from January 2022,^14^ a RAT (probable). Only patients with infections having a specimen collection date within 28 days before to 3 days after ED presentation were included.

Patients presenting to ED multiple times within a 28‐day period were only counted once, and the earliest ED arrival date in that period was used as the date of ED presentation.

### Outcomes

The linked database included outcome information obtained from the linked state‐wide records, so outcomes did not need to occur at the study hospital. Patient outcomes were based on the worst status of the patient at 28 days following ED arrival. The following specific worst outcomes were included for descriptive analysis: ED‐only, inpatient admission defined by the presence of an APDC, ICU admission and death. These statuses were respectively obtained from ED mode of separation in the EDDC, presence of a linked inpatient episode in the APDC for admission, hours in ICU >0 from the APDC for ICU admission, or presence of linked death registration record. ED‐only included patients with the following ED modes of separation: Departed: Treatment completed; Departed: Did not wait (DNW); and Departed: Left at own risk (LAOR). State health policy excludes treatment in the ED from being classified as an admission. Outcomes were further categorised into ‘severe’ (ICU admission or death) and ‘not severe’ (any other outcome).

### Study factors

Available variables and categories used included age (0–17, 18–29, 30–64, ≥65 years) and sex, date of arrival, date of discharge, mode of arrival (‘Ambulance’, ‘Non‐Ambulance’), triage category, specimen collection date prior to ED presentation date (yes, no), COVID‐19 period (‘pre‐Delta’, ‘Delta’, ‘Omicron’), total inpatient length of stay (LOS) in days, hours in ICU, hours on ventilation and extracorporeal membrane oxygenation (ECMO). Ambulance arrivals included State Ambulance Vehicle and Internal Ambulance/Transport.

The triage category is allocated per the Australasian Triage Scale (ATS), which is allocated as 1: Resuscitation; 2: Emergency; 3: Urgent; 4: Semi‐urgent; and 5: Non‐urgent.^15^ Due to low counts in the highest and lowest urgency categories (together representing <5% of all patients), triage category was combined into three categories: most urgent (ATS 1 and 2), somewhat (ATS 3), least urgent (ATS 4 and 5).

**Figure 1:**
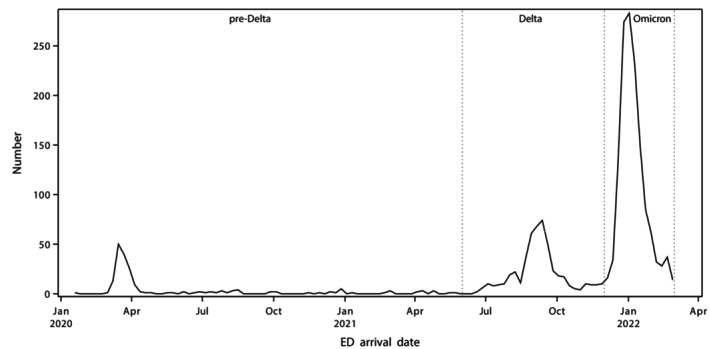
Weekly number of included ED presentations of patients with confirmed or probable COVID‐19, 1 January 2020 through 28 February 2022, and pandemic periods included. As required by the data provider, non‐zero counts <5 were rounded to the nearest 5 to avoid patient information disclosure risk.

The pre‐Delta period was 1 January 2020 to 31 May 2021, during which SARS‐CoV‐2 transmission was generally suppressed except for several contained outbreaks.^5^ The Delta period coincided with the arrival and outbreak of the Delta variant in NSW (1 June 2021 to 30 November 2021). The Omicron period included the first Omicron variant wave from 1 December 2021 to 28 February 2022 (Fig. 1).^4^

Inpatient LOS was the cumulative days spent as an inpatient counted from the earliest episode start date of any inpatient episodes commencing within 28 days of ED arrival, including admission to a short stay unit co‐located with the ED,^16^ and does not include the duration of any ED episodes. The episode start and end days were counted as whole days. ECMO was identified using procedure information recorded in the admitted patient record.

### Analysis

Logistic regression was used to estimate associations between available independent variables and a severe outcome within 28 days of initial ED arrival. Independent variables included age, sex, ambulance transport, triage category, positive COVID‐19 specimen prior to ED presentation and COVID‐19 period. Based on locally estimated scatterplot smoothing (LOESS) of the log‐odds of a severe outcome against age from the full model, age did not meet the linearity assumption for a continuous variable in logistic regression. Therefore, age was treated as a piecewise variable, with cut‐offs at 30 and 65 years, allowing the association with the outcome to be estimated per year of increase in age within each age band. The age cut‐offs were selected based on the linearity assessment, Akaike's information criterion, and clinician acceptability. Since there were no severe outcomes in persons aged <18 years, children were excluded from the model. No variables had missing values.

Data were analysed using SAS Enterprise Guide (version 9.4) and R (for regression).

### Ethics

The study was approved by the NSW Population and Health Services Research Ethics Committee (2021_ETH00070).

## Results

A total 2081 patients met the inclusion criteria, with incidence varying throughout the study period (Fig. 1). The proportion of patients acquiring COVID‐19 in Australia was lowest in the pre‐Delta COVID‐19 period (79 of 195, 40.5%), compared with 489 of 492 (99.4%) in the Delta period and 1391 of 1394 (99.8%) in the Omicron period. There were 24 (2.5%) probable cases among the 967 cases registered from 1 January through 28 February 2022 when RATs were first introduced for community use.

The mean age of all patients was 45 years (range 0–100). Mean age was lowest (35 years) in patients only with an ED episode and highest (81 years) in patients that died. Overall, 53.9% of patients arrived by ambulance, compared with 87% of patients with an ICU admission and 96% who died. Overall, just over three quarters were triaged as most (17.1%) or somewhat urgent (58.6%) on initial presentation. Two‐thirds of patients overall presented during the Omicron period and 51% of the positive COVID‐19 specimens were collected before the patient's ED arrival date (Table 1).

**TABLE 1:** Demographic and presenting characteristics of 2081 ED patients with COVID‐19 and an ED discharge diagnosis related to acute respiratory infection, by worst outcome within 28 days

Characteristic, statistic	ED‐only†, *n* = 1062	Inpatient admission, *n* = 781	ICU admission, *n* = 150	Died, *n* = 88	All patients, *n* = 2081
Age, mean (SD)	34.6 (19.7)	54.0 (25.2)	55.3 (16.1)	81.0 (9.3)	45.3 (24.6)
Age group (years), *n* (%)					
<18	163 (15.3)	64 (8.2)	0 (0.0)	0 (0.0)	227 (10.9)
18–29	317 (29.8)	90 (11.5)	7 (4.7)	0 (0.0)	414 (19.9)
30–64	487 (45.9)	318 (40.7)	95 (63.3)	7 (8.0)	907 (43.6)
≥65	95 (8.9)	309 (39.6)	48 (32.0)	81 (92.0)	533 (25.6)
Male, *n* (%)	502 (47.3)	400 (51.2)	99 (66.0)	51 (58.0)	1052 (50.6)
Ambulance transport, *n* (%)	341 (32.1)	566 (72.5)	131 (87.3)	84 (95.5)	1122 (53.9)
Triage category, *n* (%)					
Most urgent (categories 1 and 2)	95 (8.9)	165 (21.1)	60 (40.0)‡	40 (45.5)‡	356 (17.1)
Somewhat urgent (category 3)	592 (55.7)	499 (63.9)	80 (53.3)‡	50 (56.8)‡	1220 (58.6)
Least urgent (categories 4 and 5)	375 (35.3)	117 (15.0)	10 (6.7)‡	0 (0.0)‡	505 (24.3)
COVID‐19 period, *n* (%)					
Pre‐Delta	109 (10.3)	51 (6.5)	31 (20.7)	4 (4.5)	195 (9.4)
Delta	103 (9.7)	282 (36.1)	85 (56.7)	22 (25.0)	492 (23.6)
Omicron	850 (80.0)	448 (57.4)	34 (22.7)	62 (70.5)	1394 (67.0)
Specimen date before ED arrival date, *n* (%)	480 (45.2)	434 (55.6)	95 (63.3)	53 (60.2)	1062 (51.0)

† ED‐only includes patients who departed hospital after ED treatment and 31 (2.9% of ED‐only) who did not wait or who left at their own risk.

‡ The data provider required small cell counts to be masked to avoid patient information disclosure risk, so these table cells were rounded to the nearest multiple of 10.

SD, standard deviation.

### Outcomes

**Figure 2:**
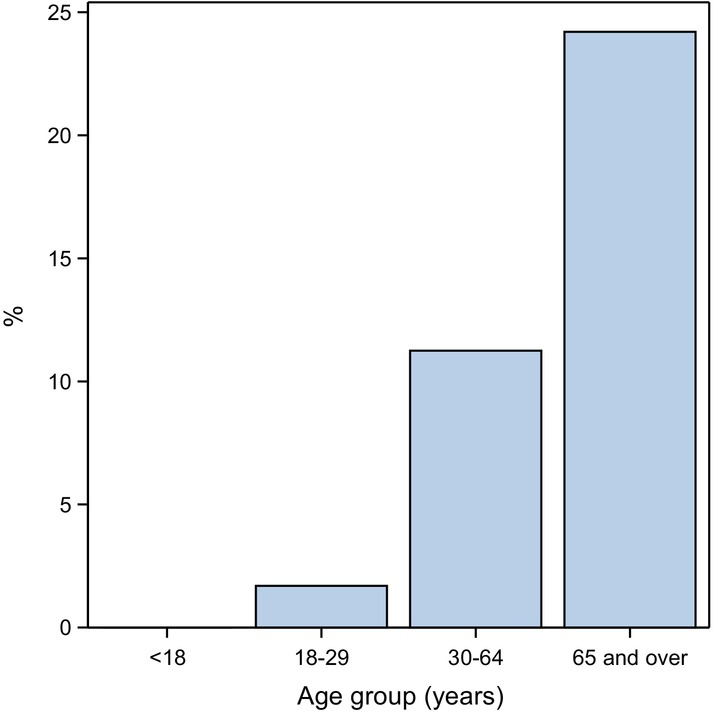
Proportion of patients experiencing a severe outcome, by age group.

A total of 1062 (51.0%) patients had a worst 28‐day outcome of ED presentation only, 781 (37.5%) had inpatient admission, 150 were admitted to ICU (7.2%) and 88 (4.2%) died within 28 days of presentation (Table 1). The 1062 patients that departed the ED included 31 (2.9%) DNW or LAOR patients. Most patients with a worst outcome of inpatient admission (*n* = 566, 72.5%), or ICU admission (*n* = 131, 87.3%), or who died (*n* = 84, 95.5%), arrived by ambulance. All 88 deaths occurred in hospital (Table 1). Twenty‐one (23.9%) patients that died had an ICU admission. Of 356 most urgent patients, 165 (46.3%) were admitted as an inpatient, ~60 (~16.9%) were admitted to ICU and ~40 (~11.2%) died within 28 days (Table 1). The proportion of patients with a severe outcome increased with age, from 0% in <18 years to 24.2% in ≥65 years (Fig. 2).

The median total inpatient LOS was 9, 17 and 12 days, respectively, for patients whose worst outcome was inpatient admission, ICU admission and death. Of patients with a worst outcome of ICU admission, 28.7% were ventilated, compared with 10.2% of those that died. Among those ventilated, median hours on ventilation were 363 and 240 h for a worst outcome of ICU admission and death, respectively. Eight (5.3% of ICU worst outcome) patients received ECMO (Table 2).

**TABLE 2:** Characteristics of 1019 patients presenting to the ED with confirmed COVID‐19 and with inpatient admission, by worst outcome, 1 January 2020 through 28 February 2022

Outcome	Inpatient admission, *n* = 781	ICU admission, *n* = 150	Died, *n* = 88
Total inpatient LOS (days), median (quartiles)†	9 (4–15)	17 (11–30)	12 (7–18)
Time in ICU (hours), median (quartiles)‡	NA	109 (61–314)	233 (111–386)
Ventilation, *n* (%)	NA	43 (28.7)	9 (10.2)
Time ventilated (hours), median (quartiles)§	NA	363 (199–605)	240 (157–284)
ECMO, *n* (%)		8 (5.3)	0 (0)

† Total length of stay (LOS) includes only any inpatient episodes commencing within 28 days of initial ED arrival for 1017 patients with inpatient admission.

‡ Time in ICU hours includes only patients admitted to ICU (*n* = 171).

§ Time ventilated includes only patients ventilated (*n* = 52).

ECMO, extracorporeal membrane oxygenation; NA, not applicable.

### Multivariable analysis

Within age groups, the rate of increase in the risk of a severe outcome varied. In 30–64‐year‐olds, the adjusted odds of a severe outcome increased by 4% (confidence interval [CI] 2–6) with each year of increase in age. The increases of 11% (95% CI −1, 29) in 18–29‐year‐olds and 2% (95% CI −1, 29) in ≥65‐year‐olds per year of age did not reach statistical significance. Male sex (adjusted OR [AOR] 1.65; CI 1.21–2.27), arriving by ambulance (AOR 2.85; CI 1.76–4.78) and having a higher triage category (most urgent: AOR 8.63; CI 4.41–18.12; somewhat urgent: AOR 3.13; CI 1.67–6.35) were associated with a severe outcome. Compared with the Omicron pandemic period, the adjusted odds of a severe outcome increased earlier in the pandemic, with the pre‐Delta period having an AOR of 6.18 (CI 3.59–10.66) and the Delta period an AOR of 3.64 (CI 2.49–5.35). After adjustment, having a COVID‐19 specimen taken prior to ED presentation was not associated with a severe outcome (Table 3).

**TABLE 3:** Results of a multivariable logistic regression model to determine independent predictors of a severe outcome within 28 days of ED presentation in 1854 adult patients with COVID‐19 and ED discharge diagnosis related to acute respiratory infection

Variable	Not severe, *n* (%)	Severe, *n* (%)	Unadjusted OR	Adjusted OR	*P*‐value
Age, years†					
18–29	407 (25.2)	7 (2.9)	1.11 (0.98, 1.28)	1.11 (0.99, 1.29)	0.109
30–64	805 (49.8)	102 (42.9)	1.05 (1.04, 1.07)	1.04 (1.02, 1.06)	<0.0001
≥65	404 (25.0)	129 (54.2)	1.01 (0.99, 1.03)	1.02 (1.00, 1.04)	0.071
Sex					
Male	783 (48.5)	150 (63.0)	1.81 (1.37, 2.41)	1.65 (1.21, 2.27)	0.002
Ambulance transport					
Yes	821 (50.8)	215 (90.3)	9.05 (5.95, 14.43)	2.85 (1.76, 4.78)	<0.0001
Triage category					
Most urgent (cat. 1 and 2)	232 (14.4)	96 (40.3)	13.88 (7.88, 26.47)	8.63 (4.41, 18.12)	<0.0001
Somewhat urgent (cat. 3)	948 (58.7)	129 (54.2)	4.56 (2.65, 8.56)	3.13 (1.67, 6.35)	0.001
Least urgent (cat. 4 and 5)	436 (27.0)	13 (5.5)	Reference		
COVID‐19 period					
Pre‐Delta	156 (9.7)	35 (14.7)	2.62 (1.70, 3.95)	6.18 (3.59, 10.66)	<0.0001
Delta	341 (21.1)	107 (45.0)	3.66 (2.71, 4.95)	3.64 (2.49, 5.35)	<0.0001
Omicron	1119 (69.2)	96 (40.3)	Reference		
Specimen date before ED arrival date					
Yes	840 (52.0)	148 (62.2)	1.52 (1.15, 2.01)	0.92 (0.66, 1.29)	0.638

† Age was included as a piecewise linear variable in each age band, so OR is for a single year increase in age within that age group. Collinearity among variables was rejected based on a maximum generalised variance inflation factor (GVIF) of 1.60.

cat., category; CI, confidence interval; OR, odds ratio.

## Discussion

During the COVID‐19 pandemic in NSW, almost half of the patients presenting to this major Australian tertiary referral ED with COVID‐19 and acute respiratory infection were admitted to hospital or died, and more than 1 in 10 had a severe outcome (ICU admission or death). There were no severe outcomes in children. Consistent with the literature,^1^, ^2^, ^17^, ^18^, ^19^, ^20^, ^21^, ^22^ outcome severity increased with age, with no severe outcomes in <18‐year‐olds rising to almost one‐quarter of ≥65‐year‐olds experiencing ICU admission or death.

When examining change in risk of a severe outcome with increasing age *within age groups*, we only found a statistically significant, independent rate of change in the 30–64‐year age group, with a 4% increase in odds per year of age. The low rate of increase within the oldest age group could reflect the effectiveness of visitor restrictions and other containment measures in residential aged care facilities combined, later, with prioritisation of COVID‐19 immunisation in aged care workers and residents, and older Australians more generally.^23^ Difficulty transferring aged care residents to hospital during the pandemic in Australia may have led to elderly patients who died being missed from our database, although death rates among aged care residents were low compared with other countries.^24^

Arrival by ambulance was also a predictor of severe outcome. While this is unsurprising, the strength of the association may have been influenced by stay‐at‐home or travel restriction public health orders that might have influenced a patient's or their carer's choice of transport to the ED.

The NSW government funded free, widespread COVID‐19 RT‐PCR testing clinics during the study period,^14^ yet a reported positive specimen was collected prior to the date of ED arrival in only around one half of patients. This finding may be biased without data on false negative specimens or RATS collected prior to ED attendance, but bias would be low because the sensitivity of RT‐PCR tests for SARS‐CoV‐2 is around 95%^25^ and RATs only became available in the final 2 months of the study and represented <3% of study patients in that period. Reasons for non‐use of community testing could include ignoring symptoms, rapid onset of severe symptoms, work exclusion if testing positive, non‐awareness of testing facilities, general practitioners referring patients to ED or concern about breaching public health orders by leaving home. In multivariable analysis, the timing of specimen collection of a positive COVID‐19 test was not associated with the odds of a severe outcome. Thus, earlier testing may not have improved the outcome of the infection.

The odds of a severe outcome were higher earlier in the pandemic, with patients presenting during the pre‐Delta period having around 6 times the odds of those presenting during the Omicron period. This may be explained by the relative severity of variants, the effectiveness of the vaccine (from February 2021) in preventing severe hospital outcomes^26^ and improvements in the treatment of COVID‐19 infection. There also appears to be a reduced risk of hospitalisation for Omicron compared to Delta in both vaccinated and unvaccinated people,^27^ whereas Delta variant infections may have had higher severity than the ancestral and Omicron variants.^28^

### Limitations

Limitations include the possibility of missing some COVID‐19 cases, especially early in 2020 before testing was freely available. Nevertheless, community RT‐PCR testing was quickly rolled out, and in‐hospital testing was routine.^14^ We did not have results of negative COVID‐19 tests. The study hospital may not have been completely representative of other EDs, as it served patients from NSW Special Health Accommodation (SHA) which provided temporary accommodation for returned travellers with COVID‐19 infection and for symptomatic community members unable to isolate, or who could not remain at home due to complex healthcare needs.^29^ The ED data lacked clinical observations or comorbidities, which would have allowed a more refined assessment of predictors of severe outcomes, and without ED arrival and departure times, we could not report on ED LOS.

## Conclusions

Older age, higher urgency at triage, arrival by ambulance and presenting earlier in the pandemic were associated with more severe outcomes. A vaccination strategy prioritising vulnerable populations and associated workers, along with improved treatment and changing variant severity, may have contributed to a reduced frequency over time of severe outcomes following ED presentation in Australia. Non‐pharmaceutical control measures in aged care facilities and, later, vaccination may have attenuated the increasing risk of a severe outcome with increasing age in the oldest population.

## Supporting information


**Appendix S1.** Supporting Information.

## Data Availability

The data that support the findings of this study are available from The NSW Centre for Health Record Linkage. Restrictions apply to the availability of these data, which were used under license for this study. Data are available from the author(s) with the permission of The Centre for Health Record Linkage.
